# Cerebellar Time and Relative Time: A Comparator-Based Dynamical Timing Model and its Relevance to Psychopathology and Therapies

**DOI:** 10.1007/s12311-026-01995-3

**Published:** 2026-04-14

**Authors:** Andras Hajnal, Kate Levenberg, Peter Adam, Christopher H. Chen

**Affiliations:** 1 Department of Neuroscience and Experimental Therapeutics, PennState College of Medicine, Hershey, PA USA; 2 Department of Psychiatry, Johns Hopkins School of Medicine, Baltimore, MD USA; 3 Institute for Solid State Physics and Optics, HUN-REN Wigner Research Centre for Physics, Budapest, Hungary; 4 Institute of Physics, University of Pécs, Pécs, Hungary

**Keywords:** Cerebellum, Neural Timing, Subjective Time Experience, Oscillations, Motor and Movement Disorders, Affective and Psychological Disorders

## Abstract

Time perception is fundamental to adaptive behavior, providing the scaffold for prediction, coordination, and learning. The cerebellum has long been recognized as a core hub for interval timing, yet its role extends beyond motor control to perceptual chronometry, reinforcement learning, and affective regulation. Here we introduce a novel framework, a Comparator-Based Dynamical Timing (CDT) model which describes distortions of subjective time as transformations that yield compression, dilation, and changes in temporal precision. In this account, subjective time is scaled by a gain factor κ. When κ > 1, subjective time dilates; when κ < 1, subjective time compresses. We synthesize convergent evidence from cerebellar anatomy, physiology, and computational modeling, and show how time distortions in psychiatric, neurodegenerative and neurodevelopmental disorders can be interpreted in the context of altered cerebellar temporal processing. We argue that cerebellar circuits operate in concert with cortical and basal ganglia oscillators in a comparator role, minimizing temporal deviation and maximizing precision. We propose that dysfunction across these interconnected networks contributes to distortions in subjective time perception observed in schizophrenia, bipolar disorder, depression, anxiety, post-traumatic stress disorder, autism spectrum disorder), and motor and movement disorders including Parkinson’s Disease. This framework provides a quantitative tool to predict and monitor the progression of psychiatric and neurodevelopmental/neurodegenerative disorders characterized by disrupted timing networks. Beyond diagnostic utility proposing an EEG-informed approach to track deviations in time perception, it also offers a translational platform for testing novel interventions, including non-invasive neuromodulation such as transcranial magnetic stimulation.

## Introduction

The ability to encode and predict temporal structure is fundamental to adaptive behavior: from coordinating movement to anticipating rewards or parsing speech, the brain must represent time with remarkable precision. Distortions of temporal perception are therefore not mere curiosity, but markers of the deep integration between neural chronometry and mental health. Subjective reports mirror this link, individuals with mania often describe time as racing, those with depression experience it as dragging, and people with schizophrenia report fragmented or disorganized temporal flow. These descriptions correspond with measurable behavioral impairments in interval estimation [1] and align with phenomenological accounts of disrupted continuity of self and consciousness in psychiatric disorders [2–6].

Neurophysiological studies provide converging evidence that such subjective and behavioral disruptions of time perception reflect abnormalities in brain timing mechanisms. Empirical work demonstrates that the phase of ongoing oscillations predicts visual detection and awareness [7, 8], underscoring their role in coherent perception. Disruption of these synchronization mechanisms is a recurring feature of brain disorders. For example, schizophrenia shows deficits in gamma-band and long-range synchrony [9–11], bipolar disorder exhibits abnormal gamma coherence during mania [12], and major depression is associated with altered resting-state coherence [13]. Similar abnormalities in oscillatory coupling have been reported in Autism Spectrum Disorder (ASD) [14, 15], Alzheimer’s disease [16, 17], and Parkinson’s disease, where pathological beta synchrony contributes to motor symptoms [18, 19]. Together, these findings highlight phase-locked oscillations as a central mechanism for coherent cognition, and their disruption as a shared pathophysiological signature across neuropsychiatric disorders. Furthermore, these findings support the concept of a “miscalibrated” neural clock, in which oscillatory rhythms fail to maintain proper alignment, producing distorted temporal experience.

The “communication-through-coherence” framework provides a mechanistic account of how neuronal oscillations enable flexible routing of information across distributed brain networks. According to this model, rhythmic synchronization of local field potentials aligns periods of excitability among neuronal ensembles, effectively opening and closing temporal “windows” for synaptic input. When two regions oscillate coherently, such as the prefrontal cortex and visual or parietal areas during attention [20–22] their excitability phases align, enhancing synaptic efficacy and promoting selective information transfer. At the neuronal level, this phase alignment regulates spike timing relative to the oscillatory cycle, ensuring that incoming spikes arrive during high-excitability phases and are more likely to influence downstream targets [23, 24]. At the network level, beta- and gamma-band coherence dynamically binds neuronal populations representing different stimulus or task features, forming transient communication channels that can be reconfigured as cognitive demands change [25, 26]. In this view, oscillatory coherence acts as a neural “carrier frequency” for selective attention, decision-making, and sensory integration. Importantly, disturbances in this coherence, such as aberrant beta synchrony in Parkinson’s disease or dysregulated gamma coupling in schizophrenia, disrupt these temporal communication windows, leading to failures in perceptual binding, cognitive flexibility, and timing precision.

Although the communication-through-coherence framework was initially developed primarily from neocortical recordings, converging evidence suggests that related principles may also apply to cerebellar interactions with distributed networks. In humans, cerebellar theta-band activity and cerebello-cortical coherence have been observed during visuomotor adaptation and tremor-related network states [27], consistent with task-dependent coupling between cerebellum and motor cortical regions [28]. In animal studies, Purkinje-cell activity can modulate cortico-cortical coherence [29], and disruption of inferior-olive coupling reduces complex-spike synchrony and rhythmicity[30, 31], supporting the idea that temporally coordinated cerebellar output can influence larger network dynamics. We therefore use the communication-through-coherence framework here not as a claim that all cerebellar timing is oscillation-based, but as a systems-level principle by which cerebellar timing signals may be aligned with cortical and thalamic targets.

At the mechanistic level, cortico-striatal-basal ganglia-thalamic circuits (CTX–BG) play a central role in neural timing, modulated by dopaminergic, glutamatergic, and GABAergic signaling [32–37]. While CTX–BG circuits have been the dominant focus of research on interval timing and temporal prediction, they do not operate in isolation. Increasing evidence indicates that precise temporal processing emerges from the interaction of multiple distributed networks, with striatal and thalamic computations requiring integration with parallel timing mechanisms.

In this context, the cerebellum represents a critical but often underappreciated partner. Beyond its well-established role in millisecond-level sensorimotor timing [38, 39], the cerebellum has been implicated in broader contributions to perceptual and cognitive domains of temporal prediction [40, 41. Anatomical and functional studies demonstrate reciprocal loops linking cerebellar nuclei with basal ganglia and prefrontal cortex [42–47], supporting the view that timing computations are coordinated across systems rather than confined to a single hub. Moreover, disruptions in cerebellar function produce not only motor timing deficits but also impairments in rhythm perception, predictive coding, and interval estimation [48, 49]. Importantly, such dysfunction has been increasingly implicated in psychiatric illness: cerebellar timing abnormalities have been observed in schizophrenia [50–52], and ASD [53], linking disrupted cerebellar chronometry to cognitive and affective symptoms. Thus, to fully understand the neural basis of temporal perception and its disturbance in psychiatric and neurodegenerative disorders, models of cortico-striatal–thalamic circuits must be extended to explicitly incorporate cerebellar involvement.

Two major computational frameworks have shaped thinking about neural timing by the cerebellum. The first emphasizes the cerebellum as a “timing machine,” generating precise delay lines and basis sets of temporal responses [54, 55,57, 58]. The second highlights the basal ganglia and cortical networks, which are thought to contribute more strongly to supra-second and beat-based timing, consistent with their dopaminergic dynamics and oscillatory architecture [48, 59]. A convergent view now suggests a division of labor: the cerebellum stabilizes sub-second timing with high precision, the basal ganglia accumulate evidence over longer durations, and cortical circuits integrate temporal priors and contextual demands.

What has been missing, however, is a unifying formalism for how *subjective* distortions of time perception arise across disorders. To address this gap, we propose the **Comparator-Based Dynamical Model of Timing (CDT)**, a novel framework first articulated here. In this account, subjective time is scaled by a gain factor κ, which governs dilation or compression of time perception. When κ is greater than one, subjective time dilates and events feel extended; when κ is less than one, time compresses and events seem shortened. Furthermore, temporal precision P(t) is introduced as a derived quantity that informs about deviation of the gain factor κ from its ‘normal’ value, operationally set κ = 1.

In the sections that follow, we review evidence for cerebellar timing from anatomy, physiology, computational models, and reward/aversion systems. We then detail the CDT model, showing how it integrates these findings into a unified framework extending our understanding into subjective time experience. Finally, we demonstrate how psychiatric and neurodevelopmental disorders, including schizophrenia, bipolar disorder, depression, anxiety, PTSD, ASD, and Parkinsonism, can be understood as distortions of CDT parameters. This integration provides new testable predictions about neural chronometry and points toward therapeutic targets for neuromodulation.

## The Cerebellum and Interval Timing

### THeories of Cerebellar Time

Computational models have proposed several ways in which cerebellar circuits generate timing. Recent work posits that diversity in granule-cell response latencies, sculpted by feedforward and feedback Golgi inhibition, yields a temporally distributed basis that supports interval coding. In the input layer, Golgi cells impose fast and slow inhibitory components that differentially delay or suppress granule-cell spiking during mossy-fiber trains, broadening latency dispersion and shaping temporal windows [60, 61]. Synaptic kinetics at the mossy fiber> granule-cell synapse extend EPSC time courses and enhance temporal heterogeneity, naturally supporting a reservoir of responses across the granule-cell population [54, 62]. Complementing this, others showed that metabotropic signaling in unipolar brush cells further expand the spectral basis available for downstream learning [55, 56, 62]. In cerebellum-like circuits of the electric fish granule-cell-like elements implement a temporal basis set to predict sensory consequences of actions [57, 63]. Downstream, temporally patterned granule-cell input is converted into precisely timed spiking, where synchronous Purkinje activity can entrain millisecond-locked firing in cerebellar nuclei, providing a reliable readout of interval structure [64]. Together, these studies converge on a model in which Golgi-shaped inhibition, synaptic diversity, and granule-cell heterogeneity generate a spectral reservoir of activity that downstream cerebellar circuits can decode for interval timing. These models highlight how different subsets of granule cell activity can be recruited to represent different intervals, with the breadth of the reservoir directly corresponding to the precision (P(t)) in the CDT model.

Modern views [40, 65, 66] of cerebellar timing integrate classical Marr–Albus adaptive filter theory with contemporary anatomical and physiological findings. In the adaptive filter framework [67], the mossy fiber–granule–Golgi network acts as a phase lead–lag compensator in which granule cells provide temporally diverse basis functions and Golgi cells shape those responses through feedforward, feedback, and tonic inhibition. Importantly, we do not propose that the granule–Golgi microcircuit alone accounts for the entire milliseconds-to-seconds range through a single tunable time constant. Rather, the subsecond basis appears to arise from heterogeneous synaptic kinetics, short-term plasticity, and inhibitory filtering within the input layer, while longer temporal extension likely depends on additional mechanisms, including unipolar brush cell signaling, Purkinje-cell learning dynamics, deep nuclear integration, and recurrent olivocerebellar and cerebro-cerebellar loops. In this view, seconds-range timing emerges from the nesting of multiple mechanisms with partially overlapping temporal domains, rather than from a single microcircuit operating uniformly across all timescales. This interpretation is more consistent with available experimental data showing temporally extended granule-cell responses and modulatory control of their duration [68], while also acknowledging that evidence for multi-second timing is stronger at the level of distributed circuit interactions than at the level of any single cellular element.

We propose that organizing cerebellar timing mechanisms through the lens of temporal scaling offers a more coherent framework for understanding how the cerebellum influences oscillatory systems. This perspective emphasizes how shared circuit motifs can flexibly stretch or compress internal timekeeping to match behavioral demands. Tables 1 and 2 provide a summary of cerebellar timing mechanisms examined from both anatomical microcircuit perspectives and temporal scaling principles, respectively.

**Table 1:** Cerebellar timing mechanisms based on anatomical elements

Mechanism	Description	Representative references
Inferior olive coupling	Gap-junction synchrony provides a millisecond phase reference; degraded IO decrease the precision P(t).	Llinás 2009 [69]; Bazzigaluppi et al. 2012 [70]; Wang et al. 2023 [71];
Granule cell temporal basis coding	Mossy fiber-granule cell dynamics generate temporal basis functions for P(t) control.	Medina and Mauk, 1999 [65]; Yamazaki & Tanaka 2009 [58]; D’Angelo et al. 2009 [72]; Billings et al. 2014 [73]; Barri et al., 2022 [73]; Chabrol et al., 2015 [62]; Gilmer et al., 2023 [55]; Guo et al., 2021 [56]
PF–Purkinje plasticity	LTD/LTP with eligibility traces aligns Purkinje pauses to conditioned intervals.	Medina et al. 2000 [74]; Mauk et al., 2014 [75]; Medina et el., 2001 [76]; Suvrathan et al. 2016 [77]
DCN dynamics	Purkinje pauses result in well-timed output spikes,	Sudhakar, et al. 2015 [78]; Person and Raman, 2012 [79]; Person and Raman, 2011 [64]; Wu et al., 2024 [80]
Cerebello–mesolimbic connections	Cerebellar projections to mesolimbic areas provide timing signals to reward and aversion systems.	Carta et al. 2019 [81]; Baek et al., 2022 [82]; Washburn et al., 2024 [83, 84]; Yoshida at al., 2022 [47]; Chen et al., 2023 [85]

**Table 2:** Cerebellar timing mechanisms based on time scaling mechanisms

Mechanism/scale	Key anatomical substrate	Core physiological signature	Behavioral expression	Representative evidence
Spectral/basis-set timing (ms–s)	Granule–Golgi network; parallel fibers → PkC	Heterogeneous, delayed/extended granule patterns as temporal bases	Learned delays, interval reproduction	Yamazaki & Tanaka 2009 [58]; Solinas et al., 2010 [86]; D’Angelo et al., 2011 [87]; Barri et al., 2022 [54]; Chabrol et al., 2015 [62]; Gilmer et al., 2023 [55]; Guo et al., 2021 [56]
PkC pause/ramp learning (100–1000 ms; seconds)	PkC simple-spike output; CF instructive input	Learned pause onset/offset; population ramps to reward	Eyeblink CR timing; self-timed actions; reward prediction timing	Johansson & Hesslow 2014 [88]; Jirenhed & Hesslow 2011 [89]; Jirenhed & Hesslow 2011 [90]; Garcia-Garcia et al., 2024 [91]
Climbing-fiber prediction error	IO → CF → PkC complex spikes	CS signals unexpected US delivery/omission; expected reward magnitude	Trial-by-trial timing adjustment; associative timing	Ohmae & Medina 2015 [92]; Larry et al., 2019 [93]; Garcia-Garcia et al., 2024 [91]
IO oscillation/phase reference (1–10 Hz)	Electrically coupled IO (Cx36)	Subthreshold oscillations; phase-reset; CF synchrony	Temporal coordination; precision of complex spikes	Llinás 2009 [69]; Xu et al., 2006 [94]; Torben-Nielsen et al., 2012, [95]; Bazzigaluppi 2012 [70]
Precise disinhibition of DCN	DCN (dentate/interposed/fastigial)	Pauses in PC activity	Movement onset timing; vigor; self-timed saccades	Person and Raman[79],; Person and Raman[64],; Wu et al., 2024, [80]
Duration- vs. beat-based timing	Cerebellum ↔ BG networks	Absolute interval vs. rhythmic/relative timing	Auditory timing, rhythm perception	Grube 2010 [48]; Teki 2011 [59]

**Fig. 1:**
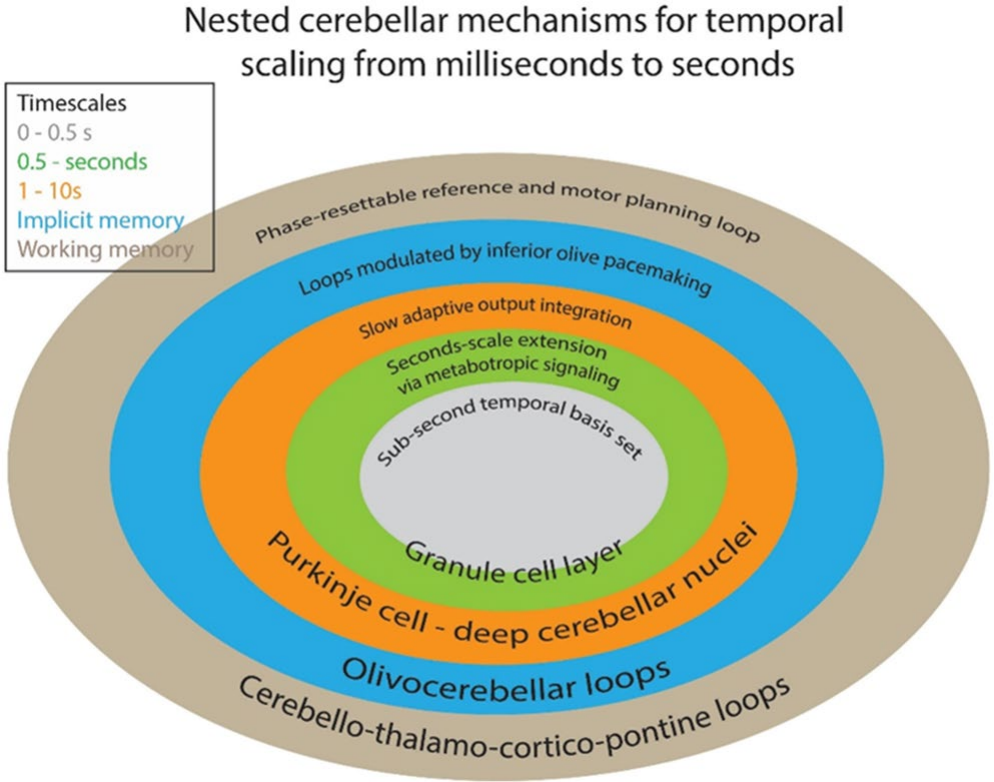
Nested cerebellar mechanisms for temporal scaling from milliseconds to seconds.Inner cerebellar input-layer circuits generate temporal basis functions through heterogeneous mossy fiber–granule cell synapses and Golgi-cell inhibition, supporting sub-second timing. Unipolar brush cells extend this basis into the seconds range through graded metabotropic signaling. Climbing-fiber teaching signals and Purkinje-cell plasticity select and weight these basis functions to produce appropriately timed pauses and ramps. Deep cerebellar nuclei integrate these learned patterns into temporally structured output, while inferior-olive oscillations and cortico-cerebellar-thalamic loops provide phase references and planning-related recurrence that scale cerebellar timing into delayed action and motor planning

Viewed from a temporal-scaling perspective, cerebellar time coding can be understood as a nested hierarchy of mechanisms (see, Fig. 1) spanning milliseconds to seconds. Here, “nested” refers not to gross anatomical compartmentalization of a morphologically uniform cerebellar cortex, but to a hierarchy of embedded physiological mechanisms in which local microcircuit dynamics are progressively extended, read out, and recalibrated by larger recurrent loops. At the core, the mossy fiber–granule cell–Golgi cell network generates sub-second temporal basis sets through heterogeneous synaptic kinetics, short-term plasticity, and inhibitory filtering, allowing granule-cell populations to represent delayed and extended temporal patterns across tens to hundreds of milliseconds [54, 62, 91]. This basis is further expanded by unipolar brush cell–dependent metabotropic signaling within the input layer, which can prolong and diversify temporal responses into the seconds range [56]. These distributed temporal signals are then selectively weighted through parallel fiber-Purkinje cell plasticity under climbing fiber teaching signals, enabling Purkinje cells to learn precisely timed pauses, ramps, and prediction signals aligned to conditioned or expected intervals [96]. At the next level, deep cerebellar nuclei integrate these learned inhibitory patterns and convert them into temporally structured output, supported by slower adaptive dynamics that extend cerebellar timing into longer behavioral windows [97]. Finally, these local cerebellar mechanisms are embedded within broader inferior olive and cerebello-thalamo-cortical loops, where phase-resettable olivary activity and recurrent interactions with thalamus and frontal cortex support predictive timing, motor planning, and working-memory-related temporal control [98–102]. In this way, cerebellar timing is not generated by a single clock, but by a layered system in which progressively larger loops extend and calibrate temporal processing across multiple timescales.

This hierarchical framework becomes clearer when examined through the lens of temporal scale, as different cerebellar regions and physiological mechanisms make partially distinct contributions across millisecond-to-second ranges. At the fastest scales (∼5–50 ms), dispersion of parallel-fiber conduction, feed-forward inhibition from basket/stellate cells, and kernel-shaping computations in Purkinje cells create narrow temporal windows-consistent with “delay-line”/“subtractive” schemes in which granule–molecular-layer dynamics sculpt precisely timed simple-spike patterns [103, 104]. Across tens to hundreds of milliseconds, adaptive simple-spike pauses learned via climbing-fiber teaching signals and/or intrinsically triggered cascades in Purkinje cells encode passage-of-time and set response latency, directly controlling deep-cerebellar-nucleus activity and conditioned movements [105–108]. Granule–Golgi loops and short-term plasticity act as tunable band-pass filters that retime population activity, enabling generalization of learned timing across nearby intervals [73]. At slower scales (hundreds of milliseconds to a few seconds), coherent subthreshold oscillations in the inferior olive, together with nucleo-olivary feedback, provide phase-resettable pacemakers that align climbing-fiber errors with expected event times, yielding scalable temporal prediction [69, 95]. Dentate/interposed ramping and rate dynamics then integrate these signals to support self-timed actions across sub- and supra-second ranges, unifying motor (eyeblink, pursuit, saccades) and non-motor (interval estimation) behaviors [109]. Functionally, the circuit multiplexes synchrony/spike-time with rate codes while learning to suppress output at non-reinforced moments, implementing a flexible temporal basis that is rescaled by context [104, 110].

### Cerebellar Time Networks and Their Embedding in Cortico–basal Ganglia Oscillatory Systems

The cerebellum does not generate temporal patterns in isolation; rather, its **internal time domains are embedded within broader oscillatory frameworks** spanning the cerebral cortex and basal ganglia. These networks share a common language of rhythmicity, although the evidence is stronger for some oscillatory regimes than others, and cerebellar oscillations have been less extensively characterized than their cortical counterparts, particularly in chronic behaving-animal preparations. By converting its timing codes into oscillatory signals, it is possible that the cerebellum both entrains and is entrained by cortical and striatal circuits, ensuring coherent temporal alignment across motor and non-motor functions. Nonetheless, recordings in awake and behaving preparations [111], together with human electrophysiology and tremor studies, support the presence of cerebellar delta-, theta-, beta-, and higher-frequency activity associated with sensorimotor processing, olivocerebellar synchrony, and cerebello-cortical coupling.

The logic behind linking cerebellar time codes to CTX–BG network oscillations can be understood as a translation chain in which intrinsic cerebellar mechanisms map naturally onto frequency bands (measurable by EEG) that dominate distributed neural rhythms, which in turn organize specific classes of behavior. At the millisecond range (5–50 ms), dispersion of parallel fiber conduction and feed-forward inhibition shape Purkinje cell responses into tightly defined temporal kernels [58, 104]. These windows align with the gamma band (30–80 Hz) in cortical and striatal circuits, where cycles of ~ 10–30 ms support perceptual binding and rapid motor updating [11]. At sub-second scales (100–500 ms), climbing-fiber-evoked pauses in Purkinje simple spikes and generate rhythms that couple to theta–beta activity (4–20 Hz), a frequency range central in CTX–BG loops for coordinating motor preparation, sequencing, and working memory [105, 112]. At longer scales of hundreds of milliseconds to several seconds, inferior olive neurons oscillate at 1–10 Hz and are stabilized by nucleo-olivary loops [69]. These pacemaker dynamics resonate with theta–delta activity in hippocampal, frontal, and basal ganglia networks, which underlie predictive timing, interval estimation, and reinforcement learning [113–115]. Taken together, cerebellar time codes—from millisecond kernels to multi-second oscillators-embed into CTX–BG rhythms by phase alignment, allowing the cerebellum to act as a flexible temporal bridge. This multiscale embedding ensures that motor outputs and cognitive operations are synchronized to the correct temporal context, maintaining fidelity of behavior across diverse timescales.

The cerebellum’s role in timing extends beyond generating precise temporal patterns; it also functions as a comparator, continuously evaluating predicted versus perceived outcomes. This concept is rooted in the most influential comparative models derived from cerebellum-like structures in weakly electric fish, where parallel fiber inputs encode contextual predictions and climbing fibers convey sensory feedback, enabling error-driven plasticity to align internal models with external reality [116, 117]. By extending this principle to mammalian cerebellum, time perception can be understood as a dynamic calibration process: cortical networks generate predictive temporal scaffolds (priors about interval duration, rhythmic regularity, or expected phase), while cerebellar circuits compare these predictions to actual sensory and motor feedback arriving via mossy and climbing fibers. One recent study has provided further support for this theory [91]. Discrepancies between expected and observed timing yield error signals that update Purkinje cell outputs, refining the temporal accuracy of cortical and basal ganglia oscillations.

Within this framework, the cerebellum not only produces subsecond timing kernels but also ensures alignment between predicted cortical time and experienced sensory time. This comparator function explains why cerebellar disruption impairs the fidelity of millisecond-scale prediction errors, leading to distortions in synchronization, interval reproduction, and anticipatory motor control. Conversely, intact cerebellar mechanisms allow flexible recalibration when external rhythms shift or when cortical priors are unreliable, thereby maintaining coherent integration of time across sensory, motor, and cognitive domains.

### Reward- and Aversion-related Signals

**The cerebellum’s role in timing is not limited to motor learning.** Increasing evidence implicates it in reinforcement learning and affective prediction. Climbing fibers have been shown to carry reward-prediction error signals, responding strongly to unexpected rewards and decreasing activity when expected rewards are omitted [92]. Purkinje cells in mice receive climbing fiber input that encodes both positive and negative reward prediction errors, further extending the instructive role of this system into motivational domains [118]. At the systems level, the dentate nucleus projects directly to the ventral tegmental area and nucleus accumbens, where it influences dopamine signaling and reinforcement learning [81, 82, 84]. The discovery of Purkinje→parabrachial and Purkinje→brainstem outputs add further channels through which cerebellar timing signals may be relayed into forebrain motivational circuits [85, 119]. These outputs provide convergent pathways for linking cerebellar chronometry to dopamine regulation, social behavior, and affective state.

The same circuits also support aversive learning. Classical eyeblink conditioning demonstrates how Purkinje cell pauses and deep nuclear disinhibition encode precisely the interval between conditioned and unconditioned aversive stimuli. Cerebellar projections to the amygdala and periaqueductal gray further highlight its role in defensive learning and fear conditioning [120].

In summary, the cerebellum provides both structural substrates and computational principles for interval timing. Its anatomy creates convergent and precisely recoded inputs; its physiology generates phase-locked reference signals, predictive pauses, and rebound spikes; and its computational strategies span adaptive filtering, spectral timing, and Bayesian integration. Recent advances underscore that cerebellar timing is broadcast widely through multiple efferent channels, including direct Purkinje outputs, parabrachial relays, basal ganglia modulation, and mesolimbic projections.

## The Comparator-Based Dynamical Model of Timing (CDT)

Neural systems can generate subjective time distortions in which events feel compressed, elongated, or unstable. Subjective time is not absolute but relative to the neural states of distributed circuits [121]. Subjective experience of time depends on oscillatory frequency, neurotransmitter tone, coupling strength, and plasticity across cerebellar, basal ganglia, and cortical networks. Here we propose the Comparator-Based Dynamical Model of Timing (CDT) which quantifies the connection between subjective and objective time and describes the evolution of the deviation.

### Formal Description and Multi-parameter Structure

The connection between the subjective ($\:\varDelta\:{t}^{\left(s\right)}$) and the objective ($\:\varDelta\:t$) time interval elapsed between close events can be described by the expression:

1 $$\:\varDelta\:{t}^{\left(s\right)}=\kappa\:\left(t\right)\cdot\:\varDelta\:t$$

Here $\:\kappa\:\left(t\right)$ is the time dependent gain factor which quantifies the ratio between subjective and objective time. When $\:\kappa\:\left(t\right)$ exceeds one, subjective time dilates, and events are experienced as lasting longer than they physically do. When $\:\kappa\:\left(t\right)$ is less than one, subjective time compresses, and events are perceived as shorter. This general expression is valid for the period times $\:{\tau\:}_{p}^{\left(s\right)}$and $\:{\tau\:}_{p}$ of periodic clock signals determining the subjective and the objective time, respectively:

2 $$\:{\tau\:}_{p}^{\left(s\right)}\left(t\right)=\kappa\:\left(t\right)\cdot\:{\tau\:}_{p}$$

The difference between subjective and objective time can be characterized by the absolute deviation $\:{D}_{a}\left(t\right)$ or the relative deviation $\:{D}_{r}\left(t\right)$ of these period times. These quantities are defined as

3 $$\:{D}_{a}\left(t\right)={\tau\:}_{p}^{\left(s\right)}\left(t\right)-{\tau\:}_{p}\:,$$

4 $$\:{D}_{r}\left(t\right)=\frac{{\tau\:}_{p}^{\left(s\right)}\left(t\right)-{\tau\:}_{p}}{{\tau\:}_{p}}=\kappa\:\left(t\right)-1.$$

We note that the reciprocal of the absolute value of the relative deviation $\:{|D}_{r}\left(t\right)|$ can be considered as a quantity characterizing the precision P(t) of the timing that is

5 $$\:P\left(t\right)=\frac{1}{{|D}_{r}\left(t\right)|\:}$$.

Higher values of *P(t)* indicate sharper precision while lower values of it reflect less reliable timing.

The regulatory mechanism of the brain strives to restore the objective timing that is it adjusts $\:{\tau\:}_{p}^{\left(s\right)}\left(t\right)$ so that it is equal to $\:{\tau\:}_{p}$, or equivalently, it adjusts $\:\kappa\:\left(t\right)$ so that it is equal to one. For finding a proper equation that describes the restoring dynamics it is plausible to assume the restoring force is proportional to the absolute deviation of the period times. On the other hand, the restoring dynamics should be smooth in order to avoid oscillatory behavior of $\:{\tau\:}_{p}^{\left(s\right)}$ around $\:{\tau\:}_{p}$. Accordingly, the dynamics of the relative deviation $\:{D}_{r}\left(t\right)$ can be described by the following differential equation:

6 $$\:\frac{{d}^{2}{D}_{r}\left(t\right)}{{dt}^{2}}=-a{D}_{r}\left(t\right)-b\frac{d{D}_{r}\left(t\right)}{dt}$$

In this expression the first term of the right-hand side the restoring force and the second term describes a damping force which ensures the smooth restoring of the timing. This latter term decreases the restoring force proportionally with the velocity of the changing of the absolute deviation $\:{D}_{r}\left(t\right)$. In this CDT model of Eq. (6) the positive parameters *a* and *b* are determined by the time regulatory mechanism of the brain in which the cerebellum works as a comparator of the subjective and the objective timing. The actual values of these parameters can be measured in a medical experiment which monitors the evolution of the subjective time at a volunteer patient. We note the same differential equation as (6) describes the evolution of the absolute deviation $\:{D}_{a}\left(t\right)$. The differential equation describing the evolution of the time dependent gain factor $\:\kappa\:\left(t\right)$ can be obtained from the Eq. (6) as

7 $$\:\frac{{d}^{2}\kappa\:\left(t\right)}{{dt}^{2}}=-a(\kappa\:\left(t\right)-1)-b\frac{d\kappa\:\left(t\right)}{dt}$$

**Fig. 2:**
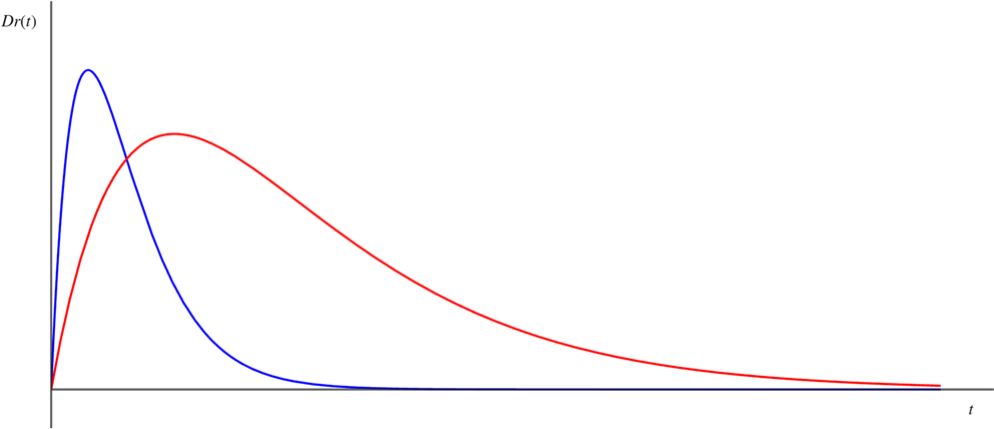
Typical evolution of the relative deviation *D*_r_(t) in the CDT model in arbitrary units. Ascending phase represents changes due to external challenges (e.g., physiological variations and pathological conditions). Descending phase evolves according to the differential equation of (6). In this phase the regulatory mechanism of the brain restores smoothly the objective timing

**Fig. 3:**
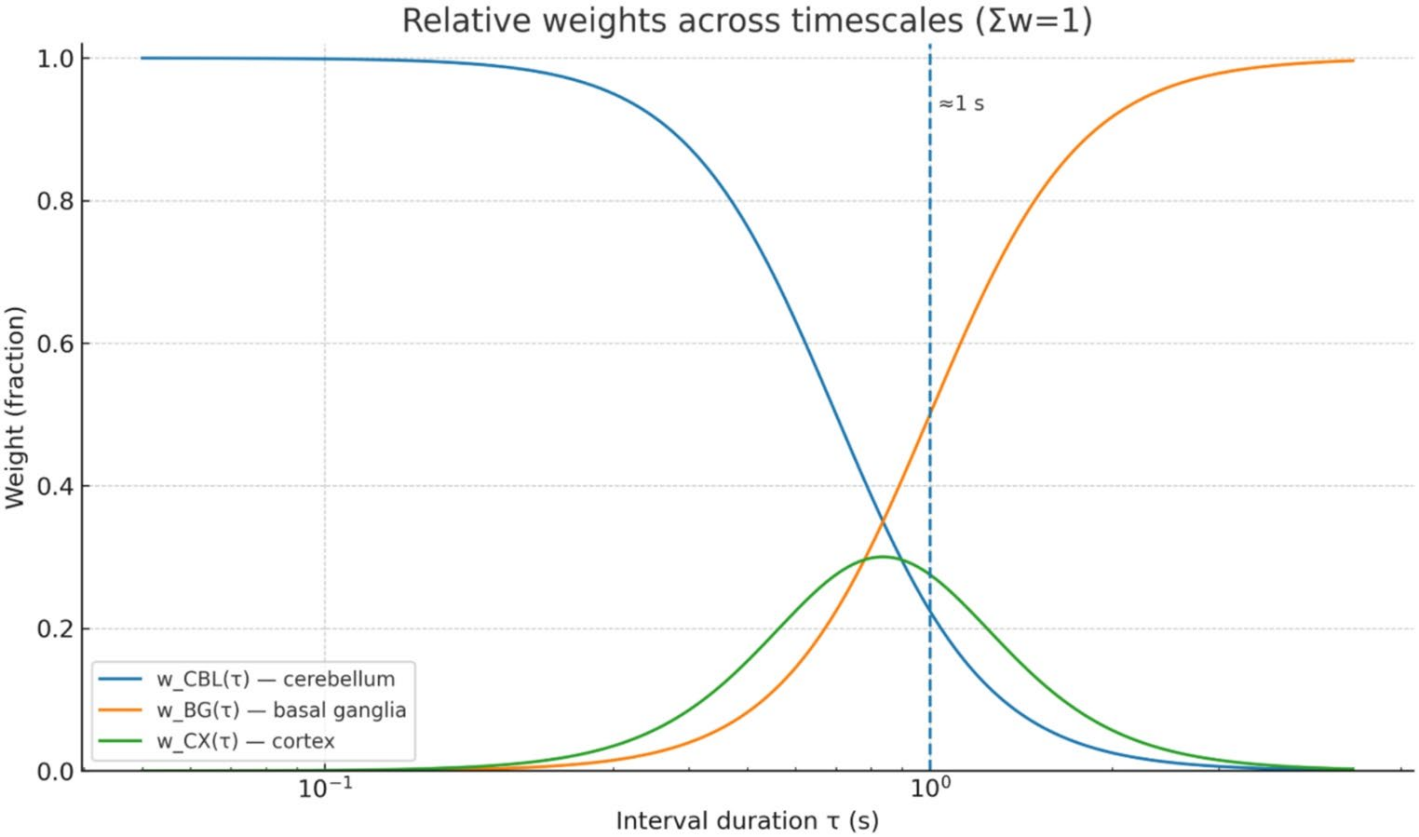
Relative weighting of cerebellar, basal ganglia, and cortical contributions across timescales. The functions w_CBL(τ), w_BG(τ), and w_CTX(τ) (normalized such that Σw = 1) describe the fractional influence of cerebellum, basal ganglia, and cortex, respectively, as a function of interval duration (τ). Subsecond timing (< 1 s) is dominated by cerebellar mechanisms, whereas supra-second intervals are increasingly governed by basal ganglia accumulation, with cortical contributions peaking at intermediate timescales where context, priors, and attentional modulation are most critical. The vertical dashed line marks the ~ 1 s transition between cerebellar- and basal ganglia–dominated regimes

A typical evolution of the relative deviation $\:{D}_{r}\left(t\right)$ obtained by solving Eq. (6) for two sets of appropriately selected values of the parameters *a* and *b* is shown in Fig. 3. In this figure the restoring of the objective timing is smooth in our dynamical model. Ascending phase represents changes due to external challenges (e.g., physiological variations and pathological conditions). In the descending phase the regulatory mechanism of the brain restores smoothly the objective timing. Note that the parameters *a* and *b* together determine the characteristics of the actual time evolution, e.g. the length of the ascending and the descending phase or the steepness of the curve. In Fig. 2, the length of these phases is longer, and the steepness is lower for the curve denoted by red line.

If one considers the actual regulatory mechanism of the brain behind this dynamical model the time dependent gain factor $\:\kappa\:\left(t\right)$ can be expressed by a multi-parameter formula that reflects the contributions of different neural systems.

8 $$\begin{array}{c}\:\kappa\:\left(t\right)=\:{\kappa\:}_{BG}^{w_{BG}}\left(t\right)\cdot{\kappa\:}_{CTX}^{w_{CTX}}\left(t\right)\:\cdot\\C\left({\kappa\:}_{CBL}\left(t\right),\:{\kappa\:}_{BG+CTX}\left(t\right)\right)\end{array}$$

This equation formalizes the gain factor $\:\kappa\:\left(t\right)$ as the joint contribution of the corresponding weighted factor of basal ganglia $\:{\kappa\:}_{BG}^{{w}_{BG}}\left(t\right)$ and the one of cortex $\:{\kappa\:}_{CTX}^{{w}_{CTX}}\left(t\right)\:$dynamically corrected by a cerebellar comparator function C$\:\left({\kappa\:}_{CBL}\left(t\right),\:{\kappa\:}_{BG+CTX}\left(t\right)\right)$. This highlights the cerebellum’s role as an error-checking mechanism, adjusting the integrated CTX–BG state according to discrepancies between expected and actual timing signals. Note, $\:\kappa\:\left(t\right)$ is the empirical time-dependent gain factor that in turn corresponds to $\:\kappa\:$ (t) gain factor in our CDT model.

Alternatively, a log-linear form can be proposed:

9 $$\begin{array}{c}\:\mathrm{ln}\kappa\:\left(t\right)=w_{BG}\cdot\:\mathrm{ln}{\kappa\:}_{BG}\left(t\right)+w_{CTX}\cdot\:\mathrm{ln}{\kappa\:}_{CTX}\left(t\right)+\\\mathrm{ln}C\left({\kappa\:}_{CBL}\left(t\right),\:{\kappa\:}_{BG+CTX}\left(t\right)\right)\end{array}$$

This log-linear form shows the same principle in additive terms: $\:\kappa\:\left(t\right)$ is expressed as a weighted sum of log-scaled BG and CTX contributions, plus a log-scaled correction term that encodes cerebellar prediction error. This representation emphasizes the interpretability of weights as linear coefficients and explicitly frames the cerebellum as a calibration system rather than a simple oscillator.

As discussed in the previous section, subsecond intervals are primarily governed by cerebellar mechanisms, supra-second intervals rely more heavily on basal ganglia accumulation, and cortical contributions modulate context, priors, and attention. This structure also captures the dissociations observed in both lesion studies and functional imaging, where cerebellar disruption impairs millisecond precision, basal ganglia disorders affect multi-second timing, and cortical damage disrupts flexible integration of temporal information [122]. See Fig. 3 for a visual summary of relative weight of sub-system across time-interval durations as it relates to time perception.

### Precision and Cerebellar Levers

**The cerebellum itself offers a set of powerful levers for stabilizing κ and maximizing P.** The inferior olive provides phase-coherent oscillations that synchronize climbing fiber signals across Purkinje cells, offering a millisecond-scale reference. Granule and Golgi cell networks expand and sculpt temporal bases, enabling Purkinje cells to extract predictive pauses through synaptic plasticity. Each of these processes maintains a stable κ, positioning the cerebellum as a precision engine of neural chronometry.

At the systems level, the CDT framework emphasizes that cerebellar computations are embedded within broader cortico–basal ganglia–cerebellar loops. Corticopontine pathways transmit α, β, and γ oscillations from cortex to mossy fibers, where they are transformed into granule cell burst patterns with heterogeneous latencies providing a temporal reservoir that downstream circuits can read out with millisecond precision [62, 64, 123–125]. Basal ganglia rhythms, especially β oscillations, influence cerebellar timing via subthalamic and thalamic relays, linking motivational and motor domains [42, 126, 127]. The inferior olive, through gap-junction synchrony, integrates these distributed inputs and imposes phase resets, with nucleo-olivary feedback providing gain control over synchrony [69, 95, 128, 129]. The cerebellar deep nuclei then broadcast these temporally structured signals to motor, cognitive, and limbic targets, embedding timing information across domains [130, 131]. The result is a low-dimensional “cerebellar time” basis that unifies cortical, basal ganglia, and cerebellar dynamics into a stable scaffold for interval and phase control [40].

**The integration of EEG with the CDT framework** could offer not only a means to quantify internal timing states but also provide a future direction to develop novel neuromodulatory clinical interventions. By continuously estimating κ, the parameter governing temporal dilation and compression, as well as estimating tempo acceleration or deceleration, one can track the shifting temporal scaffolds of neural activity in real time. When EEG rhythms such as beta bursts, theta–gamma coupling, or cerebellar-linked oscillations change systematically with behavior, these signals provide a natural readout of the CDT parameters. Such approach would effectively transform the abstract mathematics of subjective time into a clinically measurable signal [105, 132].

**From a therapeutic perspective**, this framework invites new strategies to modulate disrupted timing networks in neuropsychiatric and movement disorders. Transcranial Magnetic Stimulation (TMS) applied to the cerebellum represents a particularly promising neuromodulatory tool because the cerebellum sits at the interface of cortical and basal ganglia oscillatory loops. Stimulating the cerebellum at low frequencies in the theta or delta range can entrain or reset olivocerebellar rhythms that normally couple with cortical networks to regulate interval timing and anticipatory behavior, a mechanism relevant for anxiety and mood disorders where future-oriented processing is dysregulated [133]. Delivering stimulation in the beta range may shift cerebellar–striatal communication, potentially alleviating the excessive beta synchrony characteristic of Parkinson’s disease that underlies bradykinesia and gait-timing disturbances [134, 135]. At higher frequencies, in the gamma domain, cerebellar TMS may restore precise temporal coordination with cortical oscillations, a mechanism relevant for schizophrenia and related disorders in which gamma synchrony and perceptual binding are disrupted [11, 136]. Recent systematic reviews have highlighted the safety and preliminary efficacy of multi-session cerebellar TMS in motor and affective disorders, largely through modulation of cerebro-cerebellar connectivity and oscillatory synchrony [137]. However, evidence in schizophrenia remains mixed. A randomized controlled trial of intensive cerebellar intermittent theta-burst stimulation (iTBS) found no significant superiority of active over sham stimulation for clinical or cognitive measures [138]. Similarly, another trial reported that while vermal iTBS enhanced fronto-cerebellar resting-state connectivity, it failed to produce clear symptom improvements [139]. These findings suggest that clinical efficacy may depend on optimized stimulation protocols, particularly coil position, frequency, and phase-locking with ongoing cortical rhythms.

Within the CDT framework, cerebellar TMS may operate by adjusting comparator gain and restoring synchrony across cerebellar–cortical–basal ganglia circuits. Such modulation can recalibrate the temporal gain factor (κ → 1) and increase precision (P(t)), thereby normalizing timing-dependent behavior across motor, cognitive, and affective domains. Although protocol refinement is needed, cerebellar TMS continues to offer a translational platform for restoring synchronicity and temporal coherence in disorders from Parkinsonism to PTSD. In sum, the CDT model thus provides a mathematically grounded and biologically plausible account of subjective time and it connects microcircuit physiology, systems-level interactions, and clinical phenomenology within a single framework. In subsequent sections, we show how disorders of cerebellar and distributed circuits can be interpreted as specific distortions in κ and P(t), yielding distinct temporal signatures across psychiatric and neurological conditions, as well as informs novel therapeutic interventions.

### Psychopathologies as CDT Parameter Distortions

The CDT model provides a principled framework for understanding psychiatric and neurological disorders as systematic distortions in κ, the temporal gain parameter, and the derived parameter P(t), the precision parameter. Each disorder can be conceptualized as a unique alteration in these parameters, reflecting the underlying dysfunction of cerebellar, basal ganglia, and cortical circuits that together govern subjective time, where cerebellar circuits also exert a comparator function based on the net effects from the weight of the basal ganglia and cortical temporal signal to bring temporal distortions to zero, and subjective time to objective time (κ = 1). What follows is a brief overview of abnormalities within the CDT framework in a select major psychiatric conditions. Refer to Table 3 for hypothetical changes in specific parameters within the CDT model in various psychiatric conditions.

**Table 3:** Hypothetical changes of parameters in the proposed CDT model in psychiatric, neurodevelopmental, and neurodegenerative disorders

Condition	$\:{\kappa\:}_{\boldsymbol{C}\boldsymbol{B}\boldsymbol{L}}$	$\:{\kappa\:}_{\boldsymbol{B}\boldsymbol{G}}$	$\:{\kappa\:}_{\boldsymbol{C}\boldsymbol{T}\boldsymbol{X}}$	*P*(t)	Distortion Summary
Schizophrenia	Unstable	Normal (κ ~ 1)	Unstable	Low	Unstable, disrupted spindles/γ synchrony; impaired IO precision
Bipolar (mania)	κ < 1 (compressed)	Normal (κ ~ 1)	κ < 1	Normal/low	Compressed subsecond chronometry; accelerated cortical/striatal rhythms
Bipolar (depression)	Normal (κ ~ 1) or κ < 1	κ > 1 (dilated)	κ > 1	Low	Dilated supra-second chronometry; β-dominant BG oscillations
Major depression	Normal (κ ~ 1) or κ > 1	κ > 1 (dilated)	κ > 1	Low	Slowed subjective time; cerebello-limbic hyperconnectivity
Anxiety/PTSD	κ > 1 (threat-biased)	κ > 1 (threat-biased)	κ > 1	Low	Overestimation of aversive durations; elongated trauma recall; stress-related IO degradation
Autism spectrum disorder	Unstable	Variable	Variable	Unstable	Mismatched multisystem timing; Purkinje loss; CB–CTX dysconnectivity
Parkinsonism	κ < 1 (compensatory)	κ ≠ 1	κ > 1	Low	Supra-second dilation; β-band dominance in BG; partial CB compensation

### Schizophrenia

In schizophrenia, patients often describe fragmented or unstable temporal experiences, and behavioral studies confirm impairments in interval discrimination and temporal precision [1]. At the physiological level, reductions in spindle power (12–15 Hz), decreased gamma synchrony, and abnormal thalamocortical coupling have been widely reported [11]. These findings are consistent with NMDA receptor hypofunction and deficits in parvalbumin interneurons that destabilize oscillatory input to cerebellar circuits [140–143]. Within the CDT framework, these abnormalities decrease P(t) and destabilize κ, leading to noisy, unstable timing that align with the phenomenology of disorganized thought and temporal fragmentation.

### Bipolar Disorder

Bipolar disorder offers a striking example of bidirectional shifts in subjective time perception: manic phases are associated with overestimation or accelerated passage of time, whereas depressive states tend to produce under-estimation or a sense that time drags [144–147]. In CDT terms, mania corresponds to $\:{\kappa\:}_{{C}{B}{L}}\:$< 1, compressing subsecond chronometry through accelerated cortical and striatal rhythms, while depression corresponds to $\:{\kappa\:}_{{C}{B}{L}}$ > 1, dilating supra-second chronometry due to reduced dopaminergic tone. These alternating distortions of κ explain the characteristic oscillation between accelerated and slowed temporal experience across mood states.

### Major Depression

Major depression is frequently associated with a slowed subjective passage of time and overestimation of intervals, as supported by quantitative syntheses and clinical studies [148–150]. Structural and functional work implicates the cerebellar vermis in affective dysregulation in major depressive disorder; volumetric abnormalities of the vermis have been reported [151], and reviews/meta-analyses highlight broader cerebellar contributions to depressive symptomatology [152]. In parallel, convergent evidence indicates reduced dopaminergic function in basal ganglia/striatal circuits in major depressive disorder, consistent with motivational slowing and altered temporal processing [153, 154]. In CDT terms, these abnormalities manifest as $\:{\kappa\:}_{{B}{G}}$ > 1, producing dilated supra-second chronometry, and P(t) decreasing, which reduces temporal precision. Together, they explain the clinical experience of slowed, heavy, and blurred time in depression.

### Anxiety, Stress and PTSD

Anxiety, relating to an unpredictable event, makes time pass quicker leading to underestimating the duration of temporal intervals [155]. In contrast, post-traumatic stress disorder (PTSD) is associated with temporal overestimation [156]. Patients with PTSD consistently allocate amplified attention and judge negatively-valanced stimuli as lasting longer than neutral ones [157–159]. In stress, amygdala hyperactivity, exaggerated β-band oscillations in the basal ganglia, and HPA-axis dysregulation bias chronometry toward dilation. Acute psychosocial stress reliably produces subjective time dilation (“time slows down”) and overestimation of intervals, consistent with arousal-biased chronometry [160, 161]. Mechanistically, stress elevates HPA-axis output and cortisol, which couples to changes in EEG rhythms and central arousal [162, 163] and can directly modulate slow–fast coupling [164, 165]. In parallel, stress enhances amygdala excitatory drive and functional influence on large-scale networks, providing a biological route for salience-driven pacing of perceived time [166, 167].

Within the basal ganglia, exaggerated β-band oscillations, prominent in dopaminergic dysregulation, are linked to slowed motor/temporal updating and can bias internal clock dynamics toward dilation [18]. Together, amygdala hyperactivity, β-dominant BG states, and HPA-axis dysregulation converge on oscillatory regimes known to slow cortical updating cycles, yielding longer perceived intervals and a shift of the subjective time scale toward dilation under stress. Stress additionally disrupts inferior olive coupling, decreasing P(t) [71]. Within the CDT model, these conditions are characterized by κ > 1 combined with $\:{\kappa\:}_{{C}{B}{L}}$increasing, leading to elongated recall of trauma and overestimation of threatening durations.

In chronic anxiety and post-traumatic stress disorder (PTSD), these adaptive mechanisms become maladaptive. Heightened amygdala activity, persistent β-band synchrony in cortico-striatal loops, and dysregulated hypothalamic–pituitary–adrenal (HPA) activity bias the system toward sustained temporal dilation and reduced temporal precision P(t), degrading anticipatory control and contextual prediction [161]. In CDT terms, this corresponds to a persistent deviation κ > 1 with elevated noise in P(t), reflecting a failure of cerebellar-limbic comparator loops to restore κ ≈ 1. Such “chronometric freezing” may explain the temporal distortions and heightened expectancy that characterize trauma-related flashbacks and hypervigilance.

### Autism Spectrum Disorder (ASD)

Autism spectrum disorder (ASD) is strongly linked to cerebellar pathology, with Purkinje cell loss, vermal hypoplasia, and abnormal cerebello-cortical connectivity consistently reported [168–170]. Behaviorally, individuals with ASD show difficulties with temporal predictions in both language and music, as well as speech prosody [171]. Functional MRI and TMS studies reveal disrupted synchronization between posterior cerebellum, temporoparietal junction, and medial prefrontal cortex, regions essential for joint attention, imitation, and social anticipation [169, 172, 173]. Microstructural and functional imaging studies consistently show altered connectivity between cerebellar Crus I/II and prefrontal–striatal networks, correlating with symptom severity and social responsiveness [174].

From the CDT perspective, these findings reflect chronic desynchronization between cerebellar and cortical time bases, resulting in reduced temporal precision P(t) and fluctuating gain κ. Rather than producing uniform dilation or compression, ASD timing distortions manifest as temporal jitter, inconsistent timing of internal predictions relative to external events. This desynchrony impairs the cerebellum’s ability to compare expected versus perceived social cues, leading to reduced synchrony during conversation, gesture timing, and motor coordination. Within CDT, these patterns can be interpreted as unstable comparator calibration, where the cerebellum fails to maintain phase alignment between predicted and actual social-motor events. Therapeutically, cerebellar-targeted neuromodulation or training that enhances rhythmic entrainment may restore κ → 1 and stabilize P(t), supporting more synchronized perception and social interaction.

### Motor and Movement Disorders

Motor timing provides the most direct behavioral index of temporal regulation, and Parkinson’s disease (PD) exemplifies the breakdown of coordinated cerebellar–basal ganglia chronometry. PD patients exhibit both compressed and dilated subjective timing patterns depending on the interval range tested, indicating scale-dependent failures in the calibration of κ and P(t). At shorter intervals, PD patients often show temporal compression (κ < 1) and reduced precision P(t), consistent with excessive β-synchrony in cortico–basal ganglia loops and impaired desynchronization [175–177]. Functional and TMS studies show that cerebellar circuits attempt to compensate via feed-forward desynchronization [135, 178]. Behavioral tapping and time-counting paradigms reveal consistent under-estimation of intervals and slowed internal timekeeping, directly correlated with striatal dopamine depletion [179]. Within the CDT framework, PD represents a dual-domain dyschronometry: basal ganglia hypo- or hyper-synchrony drives deviations of.

$\:{\kappa\:}_{{B}{G}}\:$> 1, while cerebellar comparator activity is over-recruited but unable to restore κ ≈ 1. These mechanisms explain why patients with PD exhibit systematic distortions in interval timing that depend on the scale of the duration: overestimating shorter intervals while underestimating longer ones, consistent with differential effects at sub- versus supra-second timescales of timing performance [180].

In contrast, cerebellar ataxias and lesions degrade temporal precision P(t) more than the gain κ, producing variable, noisy timing rather than systematic acceleration or deceleration. Here, the comparator fails to align efferent predictions with afferent feedback, leading to both spatial dysmetria of movement and temporal dysmetria, fluctuating mismatches between predicted and actual sensory timing [181]. Collectively, these data support the view that movement disorders represent failures in comparator-based correction, where cerebellar and basal ganglia systems can no longer maintain coherent temporal calibration across distributed motor networks.

### Summary: Temporal Dysregulation Across Disorders

**Fig. 4:**
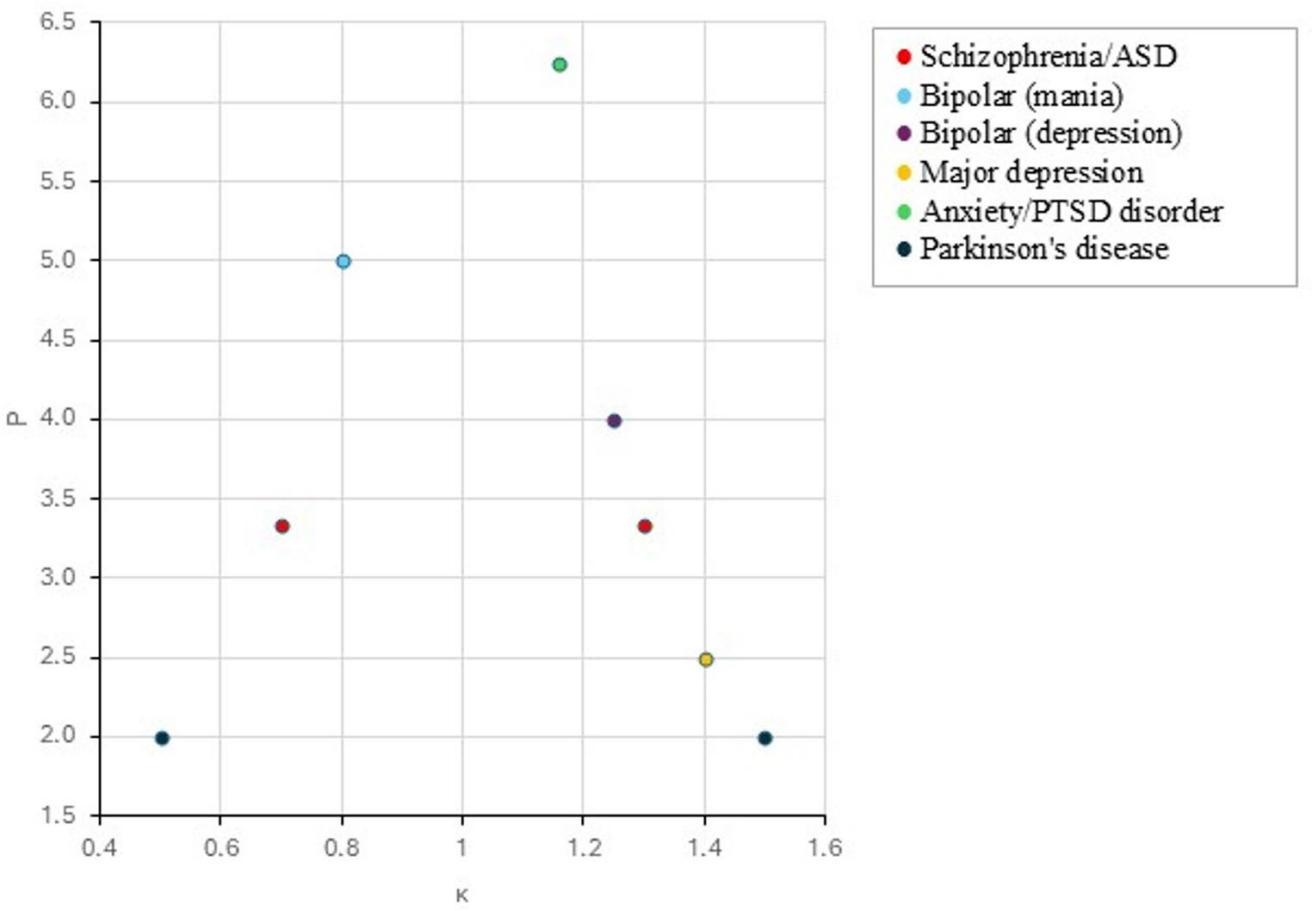
Disorders shown in the Comparator-Based Dynamical Model of Timing (CDT) plotting the gain factor $\:\kappa\:\left(t\right)$ in accordance with Eq. (8), illustrating the deviation of subjective experience of time in the patient. Schizophrenia and ASD are shown with two values as they exhibit low precision with alternating deviation of timing; bipolar disorder alternates between κ < 1 (mania, compressed) and κ > 1 (depression, dilated); major depression shows lower precision with κ > 1; anxiety/PTSD shows dilation of timing under threat; Parkinson’s disease reflects elevated κ in sub-second timing and lowered κ in supra-second timing. Larger deviation of κ from precise timing (κ = 1) yields lower precision P. Abbreviations: ASD, autism spectrum disorder; κ, temporal gain (dilation/compression); P(t), precision

Taken together, the discussions in this section support the hypothesis that psychiatric and neurological conditions can be systematically mapped onto distinct distortions in the temporal gain (κ) and precision (P(t)) parameters of the CDT model. Table 3 provides a summary on temporal dysregulation across disorders with respect to specific changes in the oscillatory systems (i.e., CB, CG, CTX) and Fig. 4 depicts an illustrative representation of the subjective experience of time.

By aligning these theoretical mappings with empirical observations, the CDT model offers a unified quantitative framework linking microcircuit-level comparator dynamics to systems-level oscillatory desynchronization and subjective distortions of temporal experience. This integrative approach reframes diverse clinical syndromes not as discrete categories but as distinct expressions of disrupted temporal homeostasis, each reflecting a specific imbalance in how the brain predicts, compares, and synchronizes time across cerebellar, basal-ganglia, and cortical networks.

### EEG-Informed CDT as a Diagnostic and Interventional Framework

We propose future theoretical work for development of an EEG-informed CDT framework that would ultimately extend beyond theory to provide both diagnostic and therapeutic potential. On the diagnostic side, we envision the model provides the capacity to continuously estimate the temporal scaling (κ) and precision (P(t)) parameters and by doing it so, these measures to serve as biomarkers of altered temporal state. Abnormal fluctuations in κ may signal disruptions in predictive coding, oscillatory synchrony, or network integration that underlie psychiatric and neurodegenerative conditions such as schizophrenia, Parkinson’s disease, or major depression. Tracking these parameters longitudinally could therefore inform prognosis, stratify patient subgroups, and monitor disease progression or treatment response. Refer to Table 4 for testable predictions linking CDT parameters, behavior, and EEG/physiology.

**Table 4:** Testable predictions linking CDT parameters, behavior, and EEG/physiology

Task	State/Condition	κ parameters	Predicted behavior	EEG/physiology readout
Eyeblink conditioning (EBC, 200–500 ms ISI)	Cerebellar lesion/degeneration	$\:{\kappa\:}_{{C}{B}{L}}$>1 (dilated) and precision decreases	Later peak CRs (conditioned responses, based on the height/amplitude ratio); poorer acquisition/precision	Reduced cerebello-thalamo-cortical drive; weaker spindle effects after learning
Predictive interception/sensorimotor synchronization (200–800 ms)	Schizophrenia (predictive timing deficit)	$\:{\kappa\:}_{{C}{B}{L}}$<1 (compression) + precision decreases; $\:{\kappa\:}_{{B}{G}}\:$, κ < 1	Early/variable responses; overestimation of pace	Fronto-cerebellar delta/theta disruption; timing variability
Temporal bisection (3 s)	Parkinson’s/hypo-dopamine; Depression (BD)	$\:{\kappa\:}_{{B}{G}}$, κ > 1 (dilated)	Longer judged durations (rightward psychometric shift)	Slower internal pace; reduced beta power/altered Individual Alpha Frequency (iIAF)
Interval reproduction (1 s)	Mania (BD)	$\:{\kappa\:\approx\:\kappa\:}_{BG}^{W}\:\cdot\:\:{\kappa\:}_{CBL}^{W}<1$	Short reproductions	iAF ↑ vs. baseline; Power Spectral Density (PSD) peaks shift up by 1/κ
Rhythmic tapping (2–4 Hz)	Cerebellar dysfunction	$\:{\kappa\:}_{CBL}$, κ > 1 and precision decreases	Greater variability (Higher Coefficient of Variation, CV), less phase locking	Impaired coupling of spindles after motor learning

On the interventional side, the same framework may be used to develop targeted strategies to actively adjust temporal dynamics (see Table 5). Because κ map onto measurable EEG rhythms, neuromodulation approaches, including transcranial magnetic stimulation (TMS), transcranial alternating current stimulation (tACS), or deep cerebellar stimulation, can be applied at rhythm-specific frequencies to shift the temporal scaffold toward more adaptive states. For example, theta-range cerebellar TMS may recalibrate anticipatory processing in anxiety, while beta- or gamma-range stimulation may counteract pathological synchrony in Parkinson’s disease or schizophrenia [182].

**Table 5:** Interventions for psychiatric and neurological conditions based on a theoretical EEG-informed CDT model

Intervention	CDT Target	EEG Biomarker Readout	Predicted Effect	Clinical Relevance
Cerebellar TMS (theta–delta)	P(t)	Cerebellar-linked theta/delta oscillations; anticipatory contingent negative variation (CNV) slope	Restores temporal anchoring for interval timing	Anxiety and mood disorders (dysregulated future-oriented processing) (Schutter & van Honk, 2009[133]
Cerebellar TMS (beta)	$\:{\kappa\:}_{{B}{G}}$(basal ganglia scaling factor)	Beta bursts; cerebellar–striatal coupling	Reduces pathological beta synchrony, improves movement initiation	Parkinson’s disease (bradykinesia, gait timing) (Koch et al., 2009 [135]; Ferrucci et al., 2015 [134]
Cerebellar TMS (gamma)	κ (network-wide temporal gain)	Theta–gamma phase amplitude coupling (PAC); cortical gamma synchrony	Restores perceptual binding and cognitive precision	Schizophrenia and psychotic disorders (disrupted gamma coherence) (Daskalakis et al., 2008 [182]; Uhlhaas & Singer, 2010 [11]
Sleep stabilization	κ (network-wide gain stability)	Cross-domain oscillatory alignment (delta, spindle-gamma coupling)	Stabilizes network-wide timing	Depression, mania, cognitive decline
Dopamine agonists	$\:{\kappa\:}_{{B}{G}}$(basal ganglia gain)	Beta/gamma power ratios, striatal beta bursts	Adjusts timing balance between exploration vs. exploitation	Parkinson’s disease, impulsivity disorders
Behavioral training	κ (temporal dilation/compression)	Task-related beta bursts, CNV slopes	Strengthens adaptive time scaling	Rehabilitation for cerebellar ataxia, motor learning deficits (Grimaldi et al., 2014[181]

The broader implication is that disorders traditionally defined by motor or cognitive symptoms can be reframed in terms of altered temporal dynamics. This opens the possibility of a new class of therapies aimed not at isolated symptoms but at restoring the brain’s intrinsic clockwork across cerebellar, cortical, and basal ganglia systems.

## Conclusions and Future Directions

Time perception is a fundamental computation of the nervous system, essential not only for motor coordination but also for cognition, affect, and social behavior. The cerebellum, long considered a specialized motor timing structure, is now recognized as a hub of interval timing across diverse domains. Its architecture and physiology make it uniquely suited to stabilize temporal precision, minimize noise, and synchronize neural chronometry with external events.

In this paper, we introduced the CDT model, a novel framework first proposed here. Building on mathematical models, the CDT model formalizes subjective time as a transformation of objective duration. A gain factor κ determines whether subjective time is dilated or compressed, while a P(t) parameter specifies the precision or noisiness of temporal estimates. Together, these parameters capture both distortions and variability of temporal experience. In its multi-parameter form, the model incorporates cerebellar, basal ganglia, and cortical contributions into the gain factor κ that dynamically shifts across timescales and task contexts.

The cerebellum emerges in this framework as a precision engine as well as a comparator for normalization of deviation between predicted and actual timing structure of all involved neural oscillators. Inferior olive synchrony provides a coherent temporal reference, granule–Golgi networks generate basis sets of temporal latencies, Purkinje cell plasticity converts these bases into predictive pauses, and deep cerebellar nuclei transform them into temporally aligned outputs. These mechanisms maximize P(t) and stabilize κ across motor, perceptual, and cognitive contexts. At the systems level, corticopontine inputs funnel cortical oscillations into cerebellar timing, while basal ganglia rhythms and dopaminergic dynamics contribute supra-second accumulation. The integration of these loops allows cerebellum, basal ganglia, and cortex to jointly regulate subjective time.

**The CDT framework generates a number of testable predictions.** If inferior olive coupling increases P(t), then manipulations that enhance gap-junction synchrony should improve temporal precision, while disruptions should decrease timing precision. If basal ganglia dopamine modulates $\:{\kappa\:}_{{B}{G}}$, then pharmacological or neuromodulatory interventions systematically bias supra-second timing toward dilation or compression. Cerebello-mesolimbic projections to the ventral tegmental area and nucleus accumbens suggest that cerebellar timing directly shapes reward chronometry, linking κ to reinforcement salience. Sleep, which restores spindle coherence and cerebello-thalamic connectivity, may recalibrate κ after perturbation, predicting that sleep deprivation should degrade temporal stability. Finally, psychiatric and neurological conditions should each exhibit distinct κ –P(t)κ fingerprints that can be measured behaviorally and with neuroimaging.

These insights have **translational implications**. By identifying how clinical time distortions map onto CDT parameters, the framework points to new biomarkers and therapeutic targets. Interventions that increase P(t), such as cerebellar neuromodulation or stimulation of inferior olive circuits, may alleviate disorders characterized by noisy timing. Treatments that adjust κ, such as dopaminergic agents or cortical rhythm modulation, may correct distorted chronometry in mood and movement disorders.

In conclusion, the CDT model provides a unified theoretical and biological framework for subjective time. By linking microcircuit mechanisms, distributed oscillatory loops, and clinical phenomenology, it offers a pathway toward a mechanistic account of temporal experience. Future research integrating systems neuroscience, computational modeling, psychiatry, and even theoretical physics may further refine this model, moving us closer to a quantitative science of subjective time—one that not only explains psychiatric distortions but also resonates with broader philosophical accounts of time as relational and emergent rather than absolute.

## Data Availability

No datasets were generated or analysed during the current study.
